# The predictive brain and the “free will” illusion

**DOI:** 10.3389/fpsyg.2013.00131

**Published:** 2013-04-30

**Authors:** Dirk De Ridder, Jan Verplaetse, Sven Vanneste

**Affiliations:** ^1^Brai^2^n, TRI and Department of Neurosurgery, University Hospital AntwerpEdegem, Belgium; ^2^The Moral Brain, Department of Legal Theory and Philosophy, University GhentGhent, Belgium; ^3^Department of Translational Neuroscience, Faculty of Medicine, University of AntwerpEdegem, Belgium

Recently a unified brain theory was proposed (Friston, 2010) attempting to explain action, perception, and learning (Friston, 2010). It is based on a predictive brain with Bayesian updating, and Andy Clark evaluates this approach in “Whatever Next? Predictive Brains, Situated Agents, and the Future of Cognitive Science.” If such a theory exists it should incorporate multiple theories applicable to brain science such as evolutionary theory (Calvin, 1987), information theory (Borst and Theunissen, 1999; Friston, 2010), thermodynamics (Kirkaldy, 1965) and also provide us with an advanced model for a better understanding of more philosophical issues such as the so-called free will problem.

The free will problem is a philosophical battle between compatibilists and incompatibilists. According to compatibilists like Hobbes, Hume, James, and Dennet, free will is not in danger if determinism is true. Free will is perfectly compatible with a deterministic working of our universe and brain. Incompatibilists disagree but differ about the conclusion to be drawn. Hard incompatibilists such as Spinoza and Laplace conclude that there is no free will because determinism is true, while soft incompatibilists like Reid, Eccles, and Penrose believe that our free will exists because determinism is false. In arguing for indeterminism incompatibilist libertarians often refer to fashionable theories such as quantum mechanics or thermodynamics which apply stochastic, non-linear models in order to describe physical processes. Nowadays these non-linear models are also applied to brain processes (Ezhov and Khrennikov, 2005), though philosophers still disagree whether this really shows that determinism is wrong and indeterminism or chance is sufficient to decide freely.

**Figure 1:**
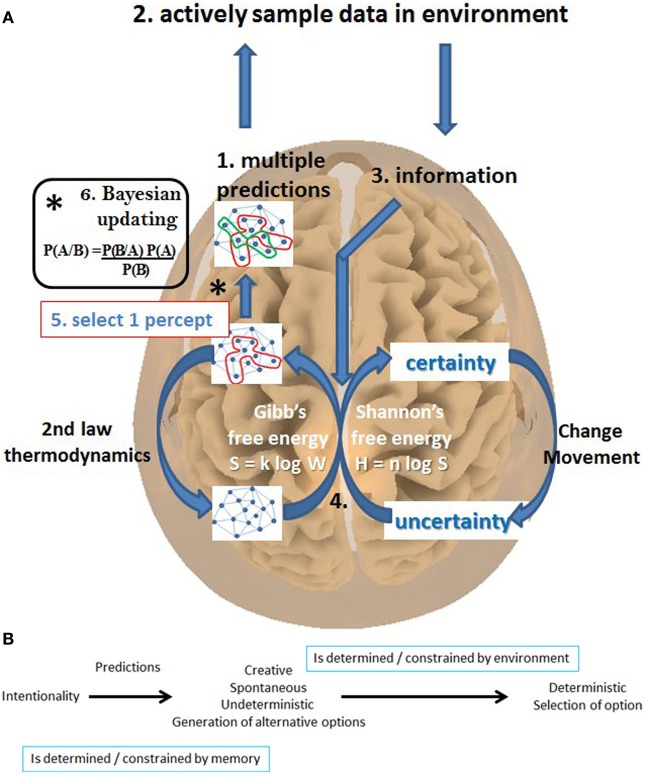
**(A)** (1) Multiple predictions are generated from memory depending on the context. (2) The environment is actively sampled/scanned for this (3) information. (4) This turns uncertainty into certainty via the use of glucose and oxygen, by creating patterns of functional connectivity in the brain. (5) One percept is selected via (6) Bayesian updating/selection. Figure modified from De Ridder et al. (2012). **(B)** Based on the goal or intention the brain generates multiple possible representations of what to expect in the environment. The representation with the smallest prediction error is selected. However the generation of representations is constrained by what is stored in memory and by the sampling of the environment.

Leaving aside this philosophical issue whether a “free will” exists or not, the authors propose a theoretical framework to explain our “experience of a free will.” This framework is based on the predictive brain concept which is not entirely new. Historically, two different models of perception have been developed, one classical view which goes back to the philosophical writings of Plato, St. Augustine, Descartes and assumes that the brain passively absorbs sensory input, processes this information, and reacts with a motor and autonomic response to these passively obtained sensory stimuli (Freeman, 2003). In contrast, a second model of perception, which goes back to Aristotle and Thomas Aquinas, stresses that the brain actively looks for the information it predicts to be present in the environment, based on an intention or goal (Freeman, 2003). The sensed information is used to adjust the initial prediction (=prior belief) to the reality of the environment, resulting in a new adapted belief about the world (posterior belief), by a mechanism known as Bayesian updating. The brain hereby tries to reduce environmental uncertainty, based on the free-energy principle (Friston, 2010). The free-energy principle states that the brain must minimize its informational (=Shannonian) free-energy, i.e., must reduce by the process of perception its uncertainty (its prediction errors) about its environment (Friston, 2010). It does so by using thermodynamic (=Gibbs) free-energy, in other words glucose and oxygen, creating transient structure in neural networks, thereby producing an emergent percept or action plan (De Ridder et al., 2012) (Figure 1A).

As completely predictable stimuli do not reduce uncertainty (there is no prediction error) they are not worthwhile of conscious processing. Unpredictable things on the other hand are not to be ignored, because it is crucial to experience them to update our understanding of the environment.

From an evolutionary point of our experience of “free will” can best be approached by the development of flexible behavioral decision making (Brembs, 2011). Predators can very easily take advantage of deterministic flight reflexes by predicting future prey behavior (Catania, 2009). The opposite, i.e., random behavior is unpredictable but highly inefficient. Thus learning mechanisms evolved to permit flexible behavior as a modification of reflexive behavioral strategies (Brembs, 2011). In order to do so, not one, but multiple representations and action patterns should be generated by the brain, as has already been proposed by von Helmholtz. He found the eye to be optically too poor for vision to be possible, and suggested vision ultimately depended on computational inference, i.e., predictions, based on assumptions and conclusions from incomplete data, relying on previous experiences. The fact that multiple predictions are generated could for example explain the Rubin vase illusion, the Necker cube and the many other stimuli studied in perceptual rivalry, even in monocular rivalry. Which percept or action plan is selected is determined by which prediction is best adapted to the environment that is actively explored (Figure 1A). In this sense, predictive selection of the fittest action plan is analogous to the concept of Darwinian selection of the fittest in natural and sexual selection in evolutionary biology, as well as to the Mendelian selection of the fittest allele in genetics and analogous the selection of the fittest quantum state in physics (Zurek, 2009). Bayesian statistics can be used to select the model with the highest updated likelihood based on environmental new information (Campbell, 2011). What all these models have in common is the fact that they describe adaptive mechanisms to an ever changing environment (Campbell, 2011).

Our evolutionary-evolved brain potential to generate multiple action plans is constrained by what is stored in memory and by what is present in the environment. Thus the feeling of a free will is an illusion, as there is likely no unlimited (=completely free) amount of representations generated, due to the inherent constraints (Figure 1B). In other words, even though neuroscience might not be able to determine whether “free will” in itself really exists, it can help unravel the mechanisms of the illusionary “experience of free will.” There are two clearly very different kinds of illusions: those with a physical cause and cognitive illusions due to misapplication of knowledge (Gregory, 1997). Cognitive illusions can be related to either specific knowledge of objects or to general knowledge embodied as rules (Gregory, 1997). Some illusions might result from the brain's tendency to use multisensory congruency as a rule or mechanism for perceptual selection (van Ee et al., 2009). For example the audio-visual McGurk effect can explain the ventriloquist illusion (Kanaya and Yokosawa, 2011). In a similar way it has been proposed that phantom sound and phantom limb illusions are related to temporal incongruence (De Ridder et al., 2011), and illusions induced by stage magic involve a perceived (simple) causal sequence which differs from the more complex real causal sequence (Kelley, 1980).
